# Maternal separation in rats induces neurobiological and behavioral changes on the maternal side

**DOI:** 10.1038/s41598-020-80087-6

**Published:** 2020-12-31

**Authors:** Ibrahim Bölükbas, Annakarina Mundorf, Nadja Freund

**Affiliations:** Division of Experimental and Molecular Psychiatry, Department of Psychiatry, Psychotherapy and Preventive Medicine, LWL University Hospital, Ruhr-University Bochum, 44780 Bochum, Germany

**Keywords:** Behavioural methods, Neuroscience, Biomarkers, Depression

## Abstract

The time after parturition is a sensitive period for mothers where they are prone to develop psychopathological symptoms. Studies investigating dams after separation from their pups (maternal separation, MS) showed that MS induces alterations similar to postpartum depression. This study aims to give further details on affected behavior and neurobiology of dams after MS. MS in rats from postnatal day 2–20 over four hours daily was performed. Upon reunion, maternal behavior, and ultrasonic vocalization (USV) of dams were measured. On the day of weaning, dams were tested for anxiety-like behavior in the elevated-plus-maze and marble burying test. Then *Morc1* mRNA in the medial prefrontal cortex and *Nr3c1* encoding the glucocorticoid receptor mRNA in the hippocampus were measured using real-time PCR to examine possible neurobiological correlates in psychopathology and social behavior. GABA and glutamate serum levels were analyzed by high-performance liquid chromatography as peripheral markers for stress-induced psychopathology. MS in dams increased maternal care towards pups even though both groups show high levels of maternal behavior even in late lactation. Furthermore, the emission of 50-kHz and 22-kHz USVs increased significantly. No differences in anxiety-like behavior were detected. MS further reduced *Morc1* but not *Nr3c1* expression. Serum GABA but not glutamate levels were significantly increased in separated dams. This study reinforces the benefit of investigating dams after MS for studying postpartum stress. Subclinical markers mainly connected to depression, namely *Morc1* and GABA, proved to be useful allowing for earlier detection of symptoms of critical postpartum stress.

## Introduction

The maternal separation (MS) paradigm is a recognized animal model to study early life stress (ELS)^1–3^. MS involves repeated daily separations of the dams from their litters^4–6^ which induces long-lasting behavioral and neurobiological changes in the offspring^7–9^. While the chronic stress exposure in this sensitive phase of early development has been widely studied, it is often ignored that the postpartum phase is also a vulnerable phase for dams or mothers making them prone to develop psychiatric disorders e.g. postpartum depression (PPD)^10^.

Only a few studies have so far focused on consequences for the dams experiencing MS^11,12^. These studies mostly investigated influences of repeated MS on maternal behavior measuring parameters such as time licking and nursing the pups or arched-back nursing^13^ and anxiety or depression-like behavior showing an increase of both, maternal care as well as anxiety and depression-like behavior after MS^14^. Anxiety-like behavior was measured using elevated-plus maze (EPM) with most studies showing decreased time spent in the open arms implying increased anxiety^12,15^. Increased depressive-like behavior was induced and demonstrated using the forced-swim test with increased immobility time^16,17^ or less sucrose intake in the sucrose consumption test^12^. Furthermore, ultrasonic vocalization (USV) emitted by the dams upon reunion can be detected as an established tool to assess affective state^18^. In rats, 50-kHz frequencies are associated with positive affect whereas 22-kHz frequencies are often reported being emitted in aversive situations such as anxiety^19^. Mother-infant disruptions have been shown to induce vocalization alterations with more 50-kHz frequencies emitted only after pups were returned^18^. Another study revealed that chronic mother-infant separation has increased the number of USVs in pups^20^.

The neuronal mechanisms that are affected by the separation and induce behavioral changes are largely unknown. Studies investigating dams after MS have found lower *Nr3c1* mRNA, encoding for the *glucocorticoid-receptor,* in the hippocampus and elevated plasma CORT levels confirming the chronic stress reaction induced by separation from the pups^12^. These studies also found that imbalances in hormones and neurotransmitters are similar to those observed in humans suffering from PPD such as altered cortisol, prolactin levels, or differences in the serotonin-system^12,16,21^.

The connection between the separation, elevated maternal care, chronic stress, and development of depressive-like symptoms is unclear but might provide valuable insights for PPD in humans.

The *MORC Family CW-Type Zinc Finger 1* (*Morc1*) gene has recently been connected to chronic stress experience during sensitive phases (so far ELS) and depression^22–24^. Besides human studies, investigating *Morc1* knockout mice revealed depressive-like behavior without any other behavioral deficits^25^.

*Morc1*′s role in ELS has been discovered in the downregulation of *Morc1* in the medial prefrontal cortex (mPFC)^22^ and using a genome-wide association study data set has been linked to major depressive disorder^22^. Investigating DNA methylation of *MORC1* in healthy humans as well as in patients showed significant associations between methylation status and depressive symptoms^23,24^. Now it would be interesting to investigate whether *Morc1* could also serve as an indicator for chronic stress exposure in the postpartum phase, especially as *Morc1* has proven to be a reliable indicator for subclinical depressive symptoms as well^23^. The mPFC is a key region for cognitive behavior, personality expression, and social behavior. Given the role of *Morc1* in mood disorders, the mPFC was analyzed regarding *Morc1* expression. On peripheral level, two key mood-regulating neurotransmitters namely glutamate and gamma-Aminobutyric acid (GABA) are getting broader attention in the pathogenesis of stress-related disorders^26–29^. These neurotransmitters are detectable in psychopathological conditions such as depression in humans^3^ due to blood–brain barrier alterations^30^. MS has demonstrated to permanently alter GABAergic and glutamate transmission and behavioral stress responses^31,32^. Several clinical studies demonstrate altered concentrations of glutamate^33^ and GABA^34^ in depressed patients’ serum, plasma, or cerebrospinal fluids. Thus, investigating glutamate and GABA serum levels in dams after MS could provide further insights but also enable translational results comparable with findings in humans. In this study, we combine a behavioral, neurochemical, and peripheral approach to characterize dams’ alterations.

## Results

### Behavioral results

#### Maternal care and USV detection

Repeated measurements for parameters over the 4 testing days showed significant results between the groups: F_(1/15)_ = 286.25; P = 0.000.

**Figure 1:**
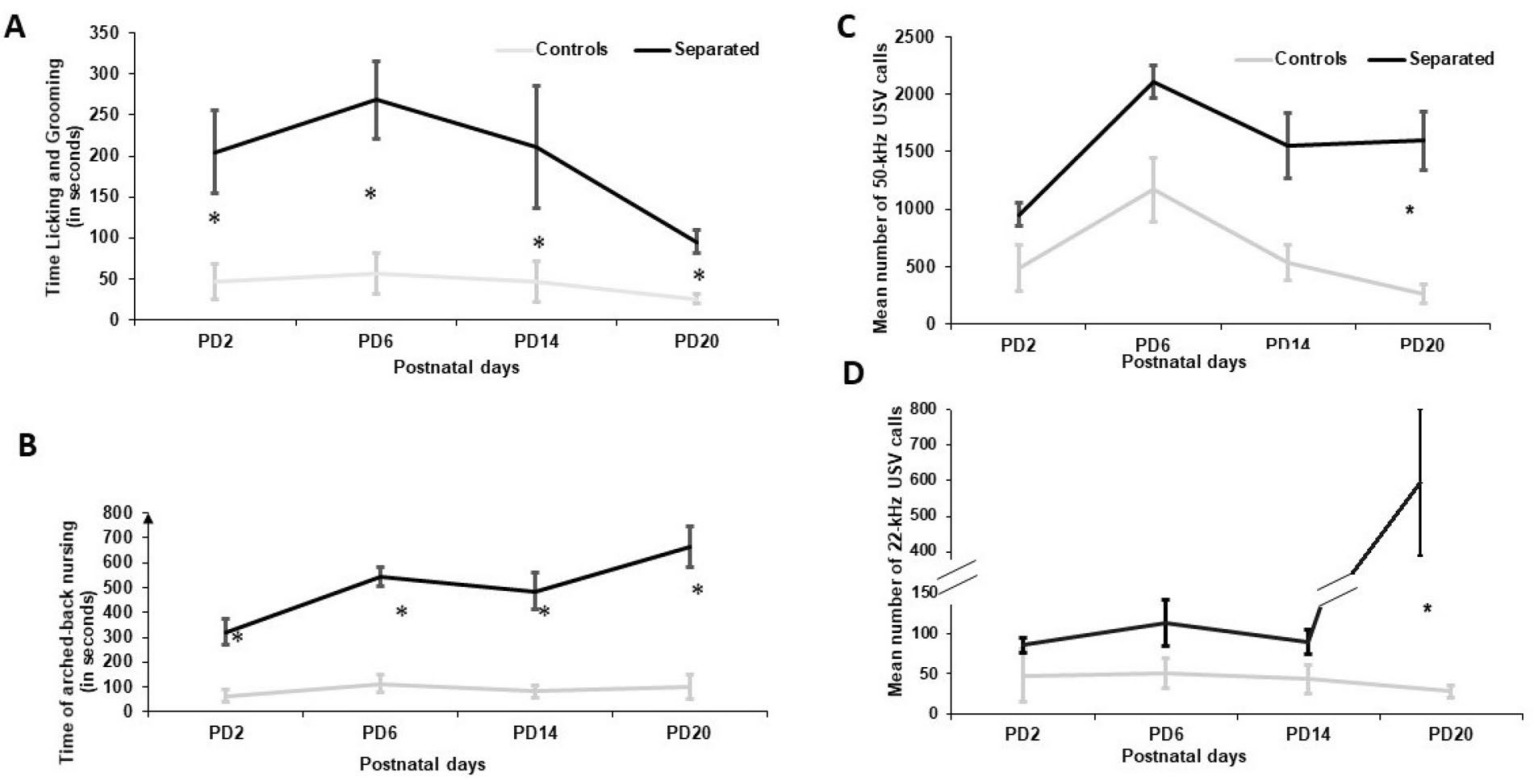
Licking and grooming (**A**) and arched-back nursing (**B**) behavior at PD2, PD6, PD14, and PD20 in separated (black) and unseparated (grey) dams. Separated dams were expressing significantly higher licking and grooming behavior. Amount of 50-kHz (**C**) and 22-kHz (**D**) frequencies calls: Separated dams (black) vocalized significantly more at 50-kHz and 22-kHz frequencies at the last day measured compared to control dams (grey). Mean number of 50-kHz and 22-kHz USV calls ± SEM are presented for n = 8 controls and n = 8 separated dams. Data were analyzed by student’s t-test.

Dams subjected to separation exhibited significantly more licking and grooming (LG) than non-separated dams at all days measured (postnatal day (PD)2: t_(14)_ = − 2.875; P = 0.01; PD6: t_(14)_ = − 3,96; P = 0.00; PD14: t_(14)_ = − 4,62; P = 0.00; PD20: t_(14)_ = − 4,72; P = 0.00, Fig.1A).

Arched-back nursing also significantly differed at the 4 time points with separated dams displaying more arched-back nursing behavior (PD2: t_(14)_ = − 4,537; P = 0.00; PD6: t_(14)_ = − 8,68; P = 0.00; PD14: t_(14)_ = − 4,537; P = 0.00; PD20: t_(14)_ = − 5.9; P = 0.00; see Fig. 1B).

Self-grooming behavior was significantly lower in separated dams at PD6, PD14 and PD20, but not at PD2 (PD2: t_(14)_ = 2.08; P = 0.06; PD6: t_(14)_ = 3.19; P = 0.01; PD14: t_(14)_ = 3,73; P = 0.00; PD20: t_(14)_ = 6,26, P = 0.00). Interestingly, the rearing time also significantly differed at PD6 and PD20 with lower scores in separated dams (PD6: t_(14)_ = 3,16, P = 0.00; PD20: t_(14)_ = 3,21; P = 0.01). Both groups did not differ in time until first pup was retrieved at any of the measured days (PD2: t_(14)_ = − 2.81; P = 0.78; PD6: t_(14)_ = − 2.22; P = 0.047; PD14: t_(14)_ = 1.02; P = 0.32; PD20: t_(14)_ = 1.10, P = 0.30). Also, the experimental groups did not differ in time until the complete litter was retrieved to the nest at any of the days investigated (PD2: t_(14)_ = − 1.61; P = 0.13; PD6: t_(14)_ = − 1.30; P = 0.22; PD14: t_(14)_ = − 1.00; P = 0.33; PD20: t_(14)_ = 1.00, P = 0.35).

Repeated measures for 50-kHz frequencies with unmodulated flat frequencies for all days measured revealed significant differences between days (F_(1/14)_ = 121.37; P = 0.000), see Fig. 1C. The 22-kHz frequencies also showed significant between groups results when all 4 days were measured (F_(1/15)_ = 13.64; P = 0.002), see Fig. 1D. For both, 22- and 50-kHz significant results were obtained at PD20 but not at the other days when using student’s t-test (for 50-kHz PD20: t_(14)_ = − 3957, P = 0.00; for 22-kHz PD20: control: t_(14)_ = − 2,65; P = 0.02).

#### Anxiety-like behavior

EPM did not reveal significant differences between groups by time spent in the open arms (control: t_(10)_ = 1.478; P = 0.17). The mean number of marbles fully covered did not differ significantly between groups (control: t_(10)_ = 0.158; P = 0.877). Test results are given in the supplementary Table 2.

### Neurobiological alterations

**Figure 2:**
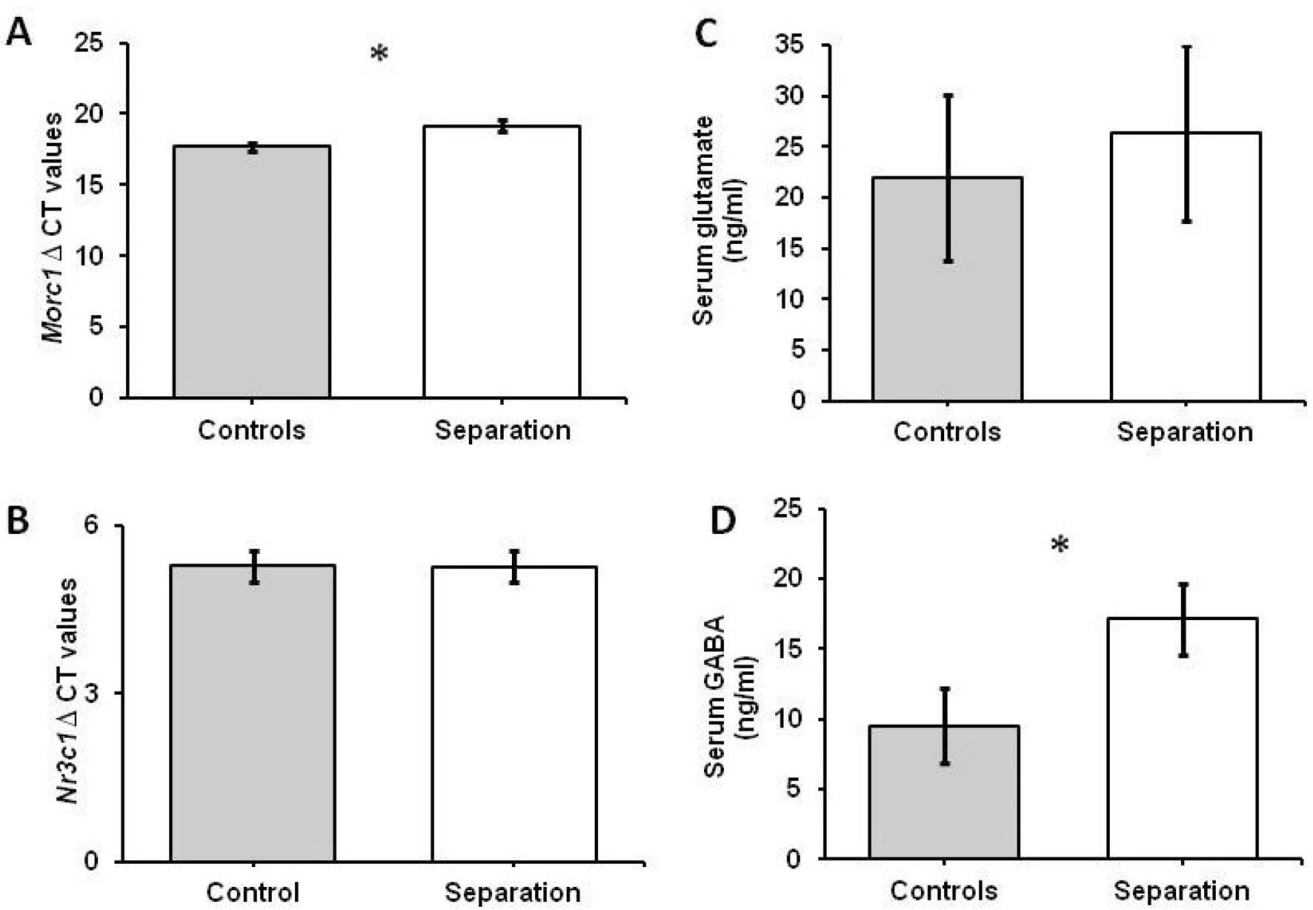
(**A**) Delta CT values of *Morc1* mRNA analyzed in mPFC brain region. Separated dams were expressing significantly lower levels of *Morc1* mRNA as shown in significant higher CT values. Means ± SEM are presented for n = 15 controls and n = 13 separated dams. *P < .01. (**B**) Delta CT values of *Nr3c1* mRNA analyzed in hippocampus brain region. Means ± SEM are presented for n = 8 controls and n = 8 separated dams. (**C**, **D**) Serum analyses of neurotransmitters glutamate for n = 14 controls and n = 15 separated dams and GABA for n = 15 controls and n = 14 separated dams after MS. Separated dams showed higher concentrations of both neurotransmitters but only GABA demonstrating significant differences. Means ± SEM are presented. Data was analyzed using a One-Way ANOVA.

Dams exposed to chronic separation from pups for four hours daily demonstrated significantly higher *Morc1* delta CT values compared to controls: F_(1, 15.5)_ = 8.19; P < 0.01 measured in the mPFC region (Fig. 2A). Values are given in supplementary Table 3.

No significant correlations were found with *Morc1* mRNA levels for the parameters with significant behavioral results (*Morc1* compared to LG: r = − 0.04; Arched-back nursing: r = 0.34; PD20: 22-kHz: r = 0.14; PD20: 50-kHz r = 0.02). Interestingly, *Morc1* levels showed significant correlations with the parameters of self-grooming, first pup retrieval and complete pup retrieval at the last day measured (*Morc1* and Self-grooming at PD20: r = − 0.594, P = 0.04; *Morc1* and first pup retrieval at PD20: r = − 0.814, P = 0.01; *Morc1* and complete pup retrieval at PD20: r = − 0.922, P = 0.01). However, after correcting for multiple testing these values did not reach significance. Furthermore, no significant correlations with *Morc1* mRNA levels and anxiety-like measures were detected (*Morc1* to marbles fully buried: r = − 0.33 and EPM time spent in open arms: r = − 0.127).

Further, we could not detect significant alterations in the hippocampus *Nr3c1* mRNA expression between groups (F_(1, 14)_ = 0.00; P = 0.99) (Fig. 2B).

### Serum GABA and glutamate

The one-Way ANOVA analysis for elevations did only reach statistical significance for GABA (F_(1,27)_ = 4.29; P = 0.048) but not for glutamate in (F_(1,27)_ = 0.14 ; P = 0.72) (Fig. 2C, D). Test results are given in supplementary Table 4.

## Discussion

Upon reunion, MS lead to overall increased maternal care and an increased number of calls emitted. It might be possible that increased maternal behavior is a manifestation of anxiety leading to exaggerated maternal care similar to overcompensation seen in humans. However, as we did not observe altered anxiety but increased 50-kHz emission, the rewarding aspect upon reunion seems to be more likely. Further studies are needed to support this claim. Testing for changes in anxiety behavior did not show significant differences between groups. However, anxiety behavior might be influenced by estrus as dams tested in the proestrus generally spent more time in the open^35^. Moreover, most studies using the long MS paradigm did not find any alterations in anxiety-like behavior in dams^36,37^ suggesting no direct effect of MS on the anxiety state of dams.

Neurobiological measurements revealed less *Morc1* mRNA in the mPFC after MS exposure but no differences in hippocampal *Nr3c1* mRNA expression. Regarding serum level analysis, significantly higher GABA levels were found in dams subjected to MS compared to controls whereas glutamate levels did not differ.

It seems that MS in rat dams during the vulnerable time of postpartum can be seen analogous to the vulnerable timeframe of pups. Accordingly, MS affects both, dams and pups and thus, it is not possible to distinguish between effects induced by the separation alone or by the altered interaction between dams and pups. The postpartum period is characterized by several physiological changes that make women prone to develop hormonal and neurochemical imbalances triggering psychiatric disorders and respective symptoms^38^.

As highlighted in other studies, we demonstrated that separated rat dams showed altered behavior towards the pups after experiencing separation stress. In this study, the separated dams increased the maternal care towards their pups upon reunion whereas controls demonstrated significantly higher levels of self-maintenance. Thus, the chronic postpartum stress and subsequent induction of maternal care might compensate and buffer the chronic stress of forced separation not only on the maternal side but also buffer stress-related responses on pups with maternal care as a possible mediating factor. Of note, our MS protocol proceeds very similarly to the protocol of other groups^4,7^. However, several differences in time and length of separations were detected^39^. Contrary to findings of other groups^40^, the dams were still maternal until the last day of testing. One interesting topic in the context of high levels of maternal behavior might be mesolimbic dopamine signaling as an up- and downregulation of this system has been shown to increase or decrease the responsiveness of maternal behavior during lactation, respectively^41^. Increased mesolimbic dopamine signaling in late lactation would be in line with increased positive-related 50-kHz USV upon reunion until weaning. This might illustrate that the reunion with pups is more rewarding after a long time of absence. Surprisingly, the 22-kHz frequencies at PD20 upon reunion were also higher in separated dams implying that separation has also led to negative mood-alterations compared to controls. Dams might be overstrained by their pups in a stress-induced mood, therefore increase the number of negatively associated affective calls as well.

On the other hand, the emitted USVs could also serve as a communication tool towards pups^42^ meaning that the increased number of calls emitted by dams upon reunion could be a response to the increased number of calls emitted by the pubs upon reunion. This could imply an affective dimension that the separation procedure is not severely limited by adaptation of dams to separation protocol but rather induces more positive and negative-related USVs in the long-term as detected in a non-invasive way of recording.

Of note, we have seen an increase in the number of 50-kHz frequencies over time which might imply the later tests were conducted with dams, the more positive emission of USVs was done by the dam upon reunion. Therefore, a constant positive affect after reunion with pups is noticeable, which is surprising given the fact that with the aging of pups, the tendency to show maternal care decreases over time. This might indicate a disruption on the behavioral level. However, the reason for the increase in maternal care in the context of the MS paradigm needs further elucidation. PD20 as the final day of testing showed significant results on both frequency ranges, but not on the first day of testing (PD2). This can show possible alterations in the affective state that are induced not immediately but rather through the repeated, chronic stress of separation. Kalinichev and colleagues also demonstrated that rat dams were more likely to emit USVs after experiencing the long MS protocol compared to controls^43^ underlying our results that rat dams were also affected by separation on mood level. The here found increase in 50-kHz calls is also in line with other groups^18^. However, one limitation of the study is that more precise analysis tools are needed to distinguish between maternal and pup USV when both are recorded simultaneously.

Gene expression downregulation of *Morc1* in separated dams compared to controls was found. Downregulation of *Morc1* either by knock-out variants^25^ or hypermethylation of *Morc1* DNA^22,23^ has been associated with depressive symptoms in other studies.

As there were no significant differences in anxiety-like behavior between groups but significant differences in *Morc1* expression it might be possible that reduced *Morc1* expression could serve as a subclinical marker indicating changes before pathological alterations manifest in behavior. This would be in line with our previous study investigating healthy young adults with subclinical depressive symptoms^23^. The chronically stressed dams did not directly demonstrate depressive-like behavior while increasing care towards pups. Correlating *Morc1* expression and maternal behavior revealed negative associations between *Morc1* and self-grooming, first pup retrieval, and complete pup retrieval at the last day measured which did not reach significance after correcting for multiple testing. Again, this might reinforce the assumption of a subclinical pathological picture induced by MS and *Morc1* as a potential regulator. Thus, the possible role of *Morc1* in subclinical or clinical depressive symptoms needs further investigation. However, *Morc1* might still be suitable for detecting chronic stress-induced neurobiological alterations.

In contrary to *Morc1,* our results on *Nr3c1* mRNA expression in the hippocampus to analyze possible acute stress reaction adaptations in the central nervous system did not reveal significant differences between groups. This has also been shown in another study with mice dams after MS^44^. Nevertheless, contradictory results with higher and lower *Nr3c1* mRNA expression have been reported in dams experiencing MS^12,45^. As *Nr3c1* expression is a possible acute stress marker^46^, and is highly influenced by the maternal care the mother expresses upon reunion with pups^47^, upregulation driven by environmental factors likely compensate neuronal adaptation of respective receptor expression.

We examined GABA und glutamate serum levels as potential peripheral markers for depression^48^ and other stress-related disorders. Studies with depressed women showed that measurements of plasma GABA levels correlated with aggressiveness levels^34^. Moreover, as there are several markers associated with stress, this study aimed at covering both central and peripheral markers to better characterize *Morc1*′s possible role in the context of stress and depression and to facilitate translation of obtained results. Thus, finding significantly higher GABA levels after MS reinforces the pathological effects of MS on dams described above. However, other studies demonstrate that the severity of depression as a chronic stress-related disorder and its symptoms are associated with glutamate levels in the blood^49,50^. As stress-related disorders have an impact on the blood–brain barrier, peripheral GABA and glutamate levels can serve as a non-invasive peripheral marker to enlighten peripheral stress-induced alterations.

The fact that some behavioral and biological pathology markers were detected (as increased maternal care and USV together with increased GABA serum levels as well as decrease *Morc1* mRNA) but no difference in others was shown (such as *Nr3c1* mRNA and glutamate serum levels together with no altered anxiety) might indicate that long term MS exposure induces subclinical depressive changes but by the time of measurement only alters some markers and not all at once.

In conclusion, analyzing maternally separated dams on the behavioral and neurobiological level can provide important information on the vulnerable time of postpartum. Investigating the maternal side might also prove necessary when analyzing the consequences of MS on pups. As maternal behavior is significantly increased after MS, this alteration might have effects on chronic stress coping of the pups. Analyzing USVs of dams upon reunion with pups proves to be an easy-to-apply and reliable tool to characterize affective states of dams. Combining behavioral and affective analyses can expand our knowledge on several chronic stress-related disorders in animal models and humans.

Besides the sensitive neonatal period, it seems that the sensitive postpartum time is vulnerable to chronic stress-induced alterations in *Morc1* expression and GABA serum levels as well. Further steps to characterize the psychopathological effects of MS on dams should include more behavioral analysis on depressive-like behavior.

## Materials and methods

### Animals

All experiments were conducted in accordance with the principles of Germany's Animal Welfare Act after approval by the animal ethics committee of the Landesamt für Natur, Umwelt und Verbraucherschutz (LANUV) in Northrhine-Westfalia. 32 timed-pregnant Sprague–Dawley rats (Charles River Laboratories, Sulzfeld, Germany) arrived at gestational days 13 to 15. Pregnant dams were housed separately in plastic cages at controlled room temperatures (22 °C) and humidity conditions (22 ± 2 °C and 55 ± 25%) with standard lighting (12 h light and 12 h of dark cycle, lights on at 11.00 pm). Dams were randomly assigned to MS or control group by a noninvolved colleague. The day of birth was considered PD0. At PD2 pups were sexed and culled to ten pups (if possible, pups were culled to five males and five female pups).

### Experimental design

In total, 32 rat dams were tested in two cohorts. In cohort 1 (N = 16) 8 rats dams underwent MS (experimental group) and 8 dams served as control group. All dams in cohort 1 were analyzed for brain and serum alterations at PD21 but did not undergo behavioral testing. In cohort 2 (N = 16) 8 rats dams underwent MS (experimental group) and 8 dams served as control group. All dams in cohort 2 underwent behavioral testing at PD21 and were further analyzed for brain and serum alterations.

All dams were anesthetized with an intraperitoneal injection of ketamine and xylazine (ratio of 10:1) and sacrificed by decapitation for serum and brain extraction.

To obtain serum probes, trunk blood was collected immediately after decapitation. We allowed the blood to clot for 20 min at room temperature. Thereafter, it was centrifuged at room temperature at 1100 g for 20 min, serum was extracted and stored at − 80 °C. Dissected brains were stored at − 80 °C for later preparation of the hippocampus and mPFC. The estrus cycle was determined according to the protocol of Marcondes and colleagues^51^ to control for cycle state influences at PD21. All dams were in the same cycling stage represented by a wide-open vagina with swollen, moist, pink tissue and by mostly nucleated and some cornified epithelial cells in the vaginal smear. This stage might either reflect proestrus^51^ or postpartum estrus^52^. Concerning the molecular analysis, the number of included animals constitutes as follows. For serum analysis, two animal samples (one animal of each group) were excluded due to technical errors and one sample per group for glutamate and GABA each. Therewith, the serum of 14 control dams and 15 MS dams was included for glutamate level analysis and serum of 15 control dams and 14 MS dams for GABA level analysis. Moreover, only the animals from cohort 1 were examined for *Nr3c1* mRNA levels. For three mPFC samples, the amount of extracted mRNA was not sufficient for *Morc1* analysis. Therefore, they were excluded.

The sample size was chosen using G*Power statistical power analysis to ensure adequate power to detect a specific effect^53^.

### Maternal separation

MS dams from cohort 1 and 2 (n = 16, experimental group) were separated from their pups for four hours daily from PD2–20^4^. They were placed in a separate adjacent cage (with free access to food and water ad libitum) in the same room with the separated pups. After the separation, both dams and pups were returned to their home cages.

In controls from cohort 1 and 2 (n = 16, control group), the dam and pups were only separated for weighing for a few minutes every 4th day.

### Maternal behavior

Maternal behavior assessment was conducted during the dark phase at around 4PM. Animals could habituate to the experimental room for 20–30 min before testing. On PD 2, 6, 14, and 20 mother–pup interactions of separated and control dams were videotaped for 15 min after reunion with pups. Two high-resolution SONY cameras were focusing on two sides of the maternal cage (one camera from the front and another from the side in approximately 20 cm distance). Maternal behavior parameters included: nursing (the dam arched-back over pups enabling access to dams’ nipples^11^, licking and grooming of pups (either head or anogenital region of pups), retrieval time of the first pup, and time to retrieve the complete litter. Furthermore, measuring time carrying pups from outside to inside the nest and time of regrouping within the nest was conducted. Parameters for self-maintenance comprised time dams spent self-grooming and rearing (with at least one paw off the ground). Maternal behavior was analyzed manually by the experimenter blinded to condition. Besides, all videos were analyzed by an independent party for subjective accuracy.

### Ultrasonic vocalizations (USVs)

USVs have proven to reflect rodents’ affective state in a non-invasive and accurate way^54^. While positive stimuli elicit shorter 50-kHz frequency ranges (33–90 kHz), negative and aversive stimuli are associated with 22-kHz frequency spans (15–32.9 kHz)^19^. To assess relative changes, each dams’ USVs were counted 15 min after reunion, respectively. Real-time recording with Batlogger M (Elekon AG, Lucerne, Switzerland) was positioned rectangular above the cage in approximately 10 cm height on PD 2, 6, 14, and 21 (Fig. 2). The minimum frequency was set at 15 kHz and the maximum frequency at 155 kHz with Volume = 2 and automatic setup trigger. Recorded USVs were analyzed using BatExplorer with FFT size of 1024 and overlap of 80% (Elekon AG, Lucerne, Switzerland). To distinguish between dams’ and pups’ USVs, non-frequency-modulated calls were regarded maternal whereas pups demonstrated atypical and U-shaped calls^18^. Only maternal calls were counted.

### Anxiety-like behavior

To assess alterations in anxiety-like behavior after MS, EPM, and marble burying^55^ were performed on the day of weaning (PD21). The EPM consisted of a plus-shaped maze approximately 50 cm in height. Two open arms (50 cm × 10 cm) and two closed arms (50 cm × 10 cm) faced in opposite directions. In a five-minute trial time, the time spent in the open and closed arms were scored and the frequency of crossings within the open or close spaces was measured (dams started in the enclosed arms). EPM was conducted during the dark phase at around 4PM followed by the marble burying test.

For the marble burying, 20 marbles were positioned symmetrically on a standard Macrolon IV cage on smoothened bedding. On three fourth of the cage area, four rows of five marbles were positioned. After 15 min, the number of marbles buried by dams (100% invisible) was counted. The number of buried marbles serves as an indirect measure for anxiety-like behavior^55^.

### Real-time quantitative polymerase chain reaction (rtPCR) of mPFC and hippocampus

The mPFC was dissected according to Watson’s The Rat Brain in Stereotaxic Coordinates at bregma 2.20 mm^56^. For the hippocampus, the whole hippocampus was dissected as defined by Watson’s rat brain atlas^56^. Isolation of RNA from the hippocampus and mPFC samples were obtained using NucleoSpin TriPrep (Macherey–Nagel, Düren, Germany) with slight modifications. For each sample, 40 μl of RNase-free water was added to obtain RNA. The concentration and quality of RNA were measured using 1 μl of the sample in NanoDrop ND-1000 Spectrophotometer (PEQLAB Biotechnologie, Erlangen, Germany).

To quantify mRNA levels, RNA was first reverse transcribed to cDNA using the High-Capacity RNA-to-cDNA Kit (Thermofisher Scientific, Darmstadt, Germany) according to the manufacturer’s protocol. Then, 60 ng of cDNA to detect *Morc1* mRNA levels in the mPFC and 60 ng of cDNA to detect *Nr3c1* mRNA levels in the hippocampus were quantified. The TaqMan hybridization with TaqMan Gene Expression Master Mix and primers using TaqMan gene expression assay for *Morc1* (Rn01474745_m1) and *Nr3c1* (Rn00561369_m1) were used. The number of target genes was normalized to the housekeeping genes *Glyceraldehyde-3-phosphate dehydrogenase* (Rn01775763_g1) and *Actin, beta* (Rn00667869_m1). The rtPCR (Applied Biosystems 7500 Fast Real-Time PCR System) reaction was quantified by the number of cycle thresholds (Delta CT method). All samples and standards were assayed in duplicates. Delta CT was calculated as delta CT = CT *Morc1*–(CT *GAPDH* + CT *beta Actin*). Both housekeeping genes have demonstrated stable expression in stress conditions^57,58^.

### High-performance liquid chromatography (HPLC) for serum GABA and glutamate

HPLC grade acetonitrile was obtained from VWR Chemicals (Langenfeld, Germany). Phtalaldehyd, 2-mercaptoethanol, glutamate, and GABA were ordered from SIGMA (Sigma-Aldrich, Taufkirchen, Germany). Furthermore, 1 M sodium hydroxide (Waldeck, Münster, Germany), methanol (Carl Roth, Karlsruhe, Germany), perchloric acid (Sigma-Aldrich, Steinheim, Germany), phosphor acid (J.T. Baker, Deventer, Netherlands) and 1 M hydrochloric acid and sodium bicarbonate from stock were used. All reagents were of the highest purity available, and solely Milli-Q deionized water was used.

Dilution series for GABA and glutamate standards were created for every new mobile phase setup before samples were assessed. For analytic identification and quantification, GABA and glutamate in serum were identified by their characteristic retention times as determined by standard injections of GABA and glutamate. The calibration curve of standard solutions was obtained from a blank, and at least four standard solutions, and the correlation coefficients of the curves were not less than 0.98. For sample preparation, 50 μl serum samples were deproteinized using an equal volume of 0.1 M perchloric acid to extract free amino acids. Serum samples and perchloric acid were centrifuged (15 min, 10,000 rpm, 4 °C) and put on ice. Then, 30 μl of supernatant was loaded into the vials. For the derivatization procedure, O-phtalaldehyde was synthesized. Samples of each serum probe were conducted in duplicates.

The serum glutamate and GABA protocol of Lee^59^ was adapted with slight modifications: Two mobile phases with 5% (pH 3.54) and 28% acetonitrile (pH 3.14) were set up separately using phosphoric acid to adjust pH (reverse phase chromatography).

Sample peak areas were measured via Labsolutions integrator system (LCsolution Version 1.24 SP1 Integration Time Program) and compared with the calibration curve standard to quantify respective concentrations. Data analysis parameters for area integration for all probes were determined with the width of 2000s, the slope of 10–30 μV/min, the drift of 0 μV/min, the doubling time (T.DBL) of 1000 min, the minimum areas/height of 1000 counts. Moreover, the integration areas were calculated with horizontal baseline, and negative peaks were rejected.

HPLC analysis was performed with the HPLC system consisted of an LC10AB chromatograph, an RF-10A XL fluorescence detector, an LC-10 AD pump, a CBM-20A system controller, a CTO-20AC oven, and a SIL-20AC autosampler system (Shimadzu, Langenfeld, Germany). All samples were injected and separated onto a reversed-phase 5µ C18 (125 mm × 4 mm) column (Multospher 100 RP 18-5µ) protected by a 30 µm pre-separation filter (Miltenyi Biotec, Germany).

### Statistical analysis

For maternal care, a second independent rater reexamined all video material. Intraclass correlation is an established statistical method to assess interrater differences of video material in subjective mother-infant relationships^60^. Only parameters showing an excellent inter-rater agreement^61^ with r =  > 0.75 were used for further analysis. Thus, the parameter carrying of pups (r = 0.59 and Cronbach’s Alpha = 0.61) was excluded.

Each maternal care parameter was analyzed separately. For the analysis, 2 treatments (separation or control) × 4 time points (PD2, PD6, PD14, and PD20) were analyzed in a two-way, repeated measures Analysis of Variance (ANOVA) for all parameters^36^ and significance was set at p < 0.00625 to control for False Discovery Rate (FDR) when analyzing multiple comparisons (P-value < 0.05 divided by 2 treatments with 4 time points). Only treatment effects, but no time point or treatment × time point interaction effects were found, and thus group differences were further analyzed by student's t-test^62^. Analogous to maternal care videos, USVs were analyzed separately for the two frequency ranges using the repeated measures ANOVA applying FDR, followed by student’s t-test. Anxiety behavior was also assessed using student’s t-test.

Data were correlated using Pearson’s correlation coefficient.

Brain and serum data were analyzed using a One-Way ANOVA with group as an independent variable (separation or control) and GABA and glutamate serum concentrations, delta CT values of *Morc1* and *Nr3c1* as dependent variables. Statistical analysis was performed using SPSS (IBM SPSS Statistics 25). Significance levels were set at p-value < 0.05.

## Supplementary Information

Supplementary tables.

## Abbreviations

ANOVA: Analysis of variance
ELS: Early life stress
EPM: Elevated-plus maze test
FDR: False discovery rate
GABA: Gamma-aminobutyric acid
HPLC: High-performance liquid chromatography
mPFC: Medial prefrontal cortex
Morc1: MORC family CW-type zinc finger 1
Nr3c1: Nuclear receptor subfamily 3 group C member 1
MS: Maternal separation
PD: Postnatal day
PPD: Postpartum depression
RT-PCR: Real-time quantitative polymerase chain reaction
USV: Ultrasonic vocalizations
