# Supplementary material for: Maternal separation in rats induces neurobiological and behavioral changes on the maternal side
Source: Sci Rep. 2020 Dec 31;10:22431. doi: 10.1038/s41598-020-80087-6 (PMC7775452; doi:10.1038/s41598-020-80087-6)
Supplement: Supplementary file 1 — Supplementary tables. [file 41598_2020_80087_MOESM1_ESM.docx]

Maternal separation in rats induces neurobiological and behavioral changes on the

maternal side

Ibrahim Bölükbas, Annakarina Mundorf and Nadja Freund

**Supplementary tables:**

Supplementary table 1: Maternal behavior on postnatal day 20 given in seconds.

| **Parameter** | **Groups** | **N** | **Mean** | **SD** |
| --- | --- | --- | --- | --- |
| PD20 - Licking and grooming | control | 8 | 25.25 | ± 15.45 |
|  | MS | 8 | 95.25 | ± 39.02 |
| PD20 - nursing | control | 8 | 100.25 | ± 141.12 |
|  | MS | 8 | 666.00 | ± 231.67 |
| PD20 - self-grooming | control | 8 | 50.50 | ± 20.69 |
|  | MS | 8 | 3.38 | ± 5.04 |
| PD20 - first pup retrieval | control | 8 | 788.38 | ± 315.72 |
|  | MS | 8 | 579.63 | ± 442.52 |
| PD20 - complete pup retrieval | control | 8 | 900.00 | ± 0.00 |
|  | MS | 8 | 815.00 | ± 240.42 |
| PD20 - rearing time | control | 8 | 235.13 | ± 122.38 |
|  | MSs | 8 | 66.50 | ± 84.04 |

Supplementary table 2: Anxiety behavior.

| **Parameter** | **Groups** | **N** | **Mean** | **SD** |
| --- | --- | --- | --- | --- |
| EPM (time spent on the open arms) in min | control | 8 | 1.98 | ± 0.50 |
|  | MS | 8 | 1.78 | ± 0.27 |
| Marble burying (number of marbles fully buried) | control | 8 | 13.75 | ± 5.09 |
|  | MS | 8 | 14.75 | ± 3.45 |

Supplementary table 3: Neurobiological Parameters are given as relative mRNA expression (delta CT values).

| **Parameter** | **Groups** | **N** | **Mean** | **SD** |
| --- | --- | --- | --- | --- |
| Relative Morc1 expression | control | 15 | 17.66 | ± 1.32 |
|  | MS | 13 | 19.15 | ± 1.44 |
| Relative Nr3c1 expression | control | 8 | 5.27 | ± 0.76 |
|  | MS | 8 | 5.26 | ± 0.76 |

Supplementary table 4: Serum Analysis given in ng/ml.

| **Parameter** | **Groups** | **N** | **Mean** | **SD** |
| --- | --- | --- | --- | --- |
| GABA levels | control | 15 | 9.52 | ± 10.29 |
|  | MS | 14 | 17.15 | ± 9.49 |
| Glutamate levels | control | 14 | 22.00 | ± 30.48 |
|  | MS | 15 | 26.38 | ± 33.52 |
